# Improving active shoulder external rotation in adults with brachial plexus birth injury and internal rotation contracture

**DOI:** 10.1016/j.xrrt.2026.100688

**Published:** 2026-02-06

**Authors:** Per Wahlström, Dag Welin, Per Nordmark

**Affiliations:** Section of Hand and Plastic Surgery, Department of Diagnostics and Intervention, Umeå University, Umeå, Sweden

**Keywords:** Brachial plexus birth injury, Shoulder external rotation, Subscapularis lengthening, Coracoidectomy, Glenoid retroversion, Humeral head alignment, Adult BPBI

## Abstract

**Background:**

Brachial plexus birth injury (BPBI) can lead to significant shoulder dysfunction, particularly internal rotation contractures that limit active external rotation (aER). This study evaluates the feasibility and outcomes of subscapular tendon step lengthening and partial coracoid resection to improve aER in adults with BPBI with no previous surgical experience. To our knowledge, no previous studies have investigated this type of surgical assessment in adults with BPBI.

**Methods:**

A retrospective analysis was conducted on 17 adult patients with BPBI. Preoperative and postoperative assessments included shoulder range of motion, glenoid and humeral head morphology, and subjective patient-reported outcomes.

**Results:**

The surgical procedure resulted in improved aER in all patients, without loss of functional internal rotation. Increased glenoid retroversion was associated with lower aER gains, as confirmed by linear regression analysis demonstrating a significant negative correlation between glenoid retroversion and postoperative improvement. Out of 17 assessed patients, 15 reported that they still would have chosen to undergo surgery based on their outcomes.

**Conclusions:**

Subscapularis tendon step lengthening and partial coracoid resection might provide a viable surgical option for improving aER in adults with BPBI with preserved glenohumeral joint morphology and internal rotation contracture. Increased glenoid retroversion negatively correlates with external rotation improvements, highlighting the need for careful patient selection.

Brachial plexus birth injury (BPBI) can lead to significant secondary musculoskeletal sequelae, with the shoulder being the most commonly and functionally affected joint.^2^^,^^5^ Internal rotation contracture, often accompanied with posterior subluxation or dislocation of the humeral head, is a frequent consequence and results in substantial limitations in shoulder function.^6^^,^^9^^,^^14^^,^^20^ One of the primary surgical challenges in managing BPBI is restoring active external rotation (aER) of the shoulder.^28^ Addressing this deficit surgically requires a thorough understanding of the anatomical and neuromuscular factors influencing the outcome.^1^^,^^3^^,^^26^ Surgical intervention is crucial not only to improve function but also to prevent further glenohumeral (GH) joint deformity.^17^ The prognosis for recovering active shoulder external rotation in individuals with internal rotation contracture secondary to BPBI is considered poor beyond a certain age.^13^^,^^15^^,^^16^^,^^21^^,^^22^^,^^27^ A key factor contributing to this unfavorable prognosis is the incomplete and ineffective reinnervation and control of the infraspinatus muscle, a primary external rotator of the shoulder.^19^^,^^24^^,^^25^ Without adequate neuromuscular function, the potential for functional recovery is severely limited. Consequently, surgical intervention with nerve repair or nerve transfers, such as the spinal accessory nerve to the infraspinatus branch of the suprascapular nerve, has been advocated to enhance the likelihood of restoring aER.^8^^,^^11^^,^^26^

However, due to variability in injury patterns and reinnervation potential, certain individuals may still benefit from targeted surgical interventions even in adulthood. This is particularly relevant in cases where the spherical morphology of the humeral head and the concave shape of the glenoid remain preserved, providing a more favorable anatomical foundation for surgical correction.^18^ In such cases, soft tissue contractures may play a more prominent role in restricting shoulder external rotation, suggesting that procedures aimed at releasing these contractures could yield meaningful improvements.

To explore this possibility, we performed subscapularis tendon lengthening in adults with BPBI who had not previously undergone surgical procedures on the affected arm. To our knowledge, this is the first study to systematically evaluate the impact of this intervention in this patient population. Patient selection was based on preserved GH joint integrity on imaging, relatively normal retroversion angles, and a distinct limitation in both aER and passive external rotation, suggesting a primary mechanical restriction rather than an irreversible neuromuscular deficit. We hypothesized that in these patients, restricted motion is not solely attributable to neurological impairment but is significantly influenced by joint stiffness and muscular contracture factors that may be modifiable through surgical intervention.

This study aims to determine the effectiveness of subscapularis release in improving external rotation in a patient population traditionally considered to have limited potential for functional recovery. Understanding the impact of surgical intervention in this group could help refine treatment strategies for adults with BPBI, particularly regarding patient selection and postoperative rehabilitation.

## Materials and methods

### Patients

Seventeen adult patients with BPBI who underwent subscapularis tendon lengthening were identified from our internal database. All patients had been diagnosed with BPBI at birth but had not undergone any surgical intervention during childhood. They represented to the health care system in adulthood because of persistent functional limitations, primarily involving restricted aER, which affected activities performed in front of the body and limited the ability to reach objects positioned high anteriorly. The indication for surgery was a limitation of aER and passive external shoulder rotation to 10° or less past neutral. Furthermore, patients were required to demonstrate at least some ability to generate aER within their existing range of motion, suggesting residual neuromuscular function that could be enhanced following contracture release.

For this study, adulthood was defined as the stage at which skeletal maturity had been achieved, as evidenced by the closure of the growth plates of the upper limb and shoulder. This definition ensures that bone growth had ceased, providing more predictable surgical outcomes in individuals with longstanding BPBI and allowing sufficient time for neurological recovery.

Because most patients presented in adulthood and detailed early medical records were unavailable, classification of nerve involvement was based on clinical function at the time of surgery. Patients with weakness limited to shoulder function, while maintaining normal elbow extension and hand function, were classified as having C5-6 involvement (Erb palsy). Those who demonstrated shoulder weakness combined with reduced active elbow and/or wrist extension strength, but preserved hand function, were classified as having C5-7 involvement (extended Erb palsy). Patients with impairment affecting shoulder, elbow, and hand function were classified as having C5-T1 involvement (global palsy). This functional categorization reflects the residual innervation patterns consistent with the originally affected nerve roots. Preoperative and postoperative clinical data were collected, including active and passive external shoulder rotation (measured in degrees with the arm adducted), abduction in the scapular plane (measured in degrees), and Modified Mallet Score (MMS). Patient age at the time of surgery was recorded.

All the affected shoulder joints had preoperatively been checked to be congruent using magnetic resonance imaging or computed tomography scan. That is, all scans were examined, and we measured, using previously described standardized methods,^10^^,^^7^^,^^29^ glenoid version, humeral head position relative to the glenoid (percentage of the humeral head anterior to the midpoint of the glenoid fossa), humeral head sphericity, and glenoid concavity, and, in scans that also included the distal humerus, humeral head version. In addition to the affected shoulders, 11 patients had bilateral radiological assessment, enabling evaluation of the glenoid and humeral head version in both the affected and contralateral unaffected arms; that is, complete radiological assessments of both shoulders and elbows. Four patients had radiological assessments of only the affected shoulder, only enabling assessment of the glenoid version in the affected shoulder. One patient had bilateral radiological assessment of shoulders, enabling glenoid version measurements in both shoulders but no humeral head measurement, and one had a unilateral assessment of the shoulder and elbow on the affected side enabling assessment of glenoid and humeral head version on the affected side only.

This allowed us to assess the glenoid version angle in all 17 affected shoulders, the contralateral shoulder in 12, the humeral head version in the affected side in 12, and in the unaffected side in 11.

### Surgical technique

Our standardized surgical technique for subscapularis lengthening in adult BPBI cases is based on previously described approaches^4^^,^^17^ and involves a deltopectoral exposure.

We perform a coracoid process resection while ensuring that the attachment of the short head of the biceps remains secured to the shortened coracoid. This is performed through a surgically induced longitudinal split in the conjoint tendon, which, after the partial coracoidectomy, is closed with size 0 braided absorbable sutures. The superior and middle GH ligament and the anterior part of the inferior GH ligament are released together with the joint capsule. This is followed by a stepwise lengthening of the subscapularis tendon. Our technique preserves the cranial half of the tendon insertion at the lesser tubercle while releasing the lower half. The released ends are then sutured together with size 0 braided absorbable sutures using 3 double-pass simple stitches, resulting in a tendon lengthening of approximately 10-15 mm compared with its prerelease length. The target with the surgical intervention is to cut the described structures with the goal of increasing aER and passive external rotation without causing GH-instability.

Postoperatively, the arm is immobilized in a brace to maintain external rotation of the shoulder for four weeks. This is followed by the initiation of movement and load-bearing exercises, with full active strengthening exercises permitted after three months.

### Patient-reported outcome

To complement clinical assessments, patients were also asked to retrospectively evaluate their experience through a standardized questionnaire. They answered 3 questions regarding the outcome of the surgical intervention:

1. “Knowing what you do now, would you still choose to undergo the surgery?” (’*Yes*'/’*no*’)
2. “Do you experience any positive effects from the surgery?”
3. “Do you experience any negative effects from the surgery?”

For the latter 2 questions, patients who responded ‘*yes’* were encouraged to specify in what way, with the option to provide multiple answers. These questions aimed to capture patient perspectives on both the benefits and drawbacks of the procedure, contributing to a more comprehensive evaluation of patient satisfaction and perceived surgical outcomes.

### Statistics

Paired two-tailed *t*-tests analyzed continuous data, while the Wilcoxon signed-rank test was applied to ordinal variables, such as Modified Mallet scores. Linear regression analysis was performed in MATLAB R2023b (MathWorks, Natick, MA, USA) using the glmfit function to assess the relationship between glenoid version and improvement in aER. Statistical significance was determined using the slope coefficient and its *P* value. In addition, a 95% prediction interval was calculated to estimate the range within which future individual observations are expected to fall, accounting for model uncertainty and natural variability.

### Ethical approval

All participants gave informed consent to participate in the study. The study was performed with ethical approval from the Swedish Ethical Review Authority (Dnr 2021-03752) and was carried out in accordance with the World Medical Association Declaration of Helsinki.

## Results

### Patient characteristics

Seventeen adult patients with BPBI underwent subscapularis tendon lengthening according to the standardized surgical technique. The mean age at the time of surgery was 25 years (range: 17-45 years). The cohort included 8 men and 9 women, with 10 patients having an affected right upper limb and 7 with a left-sided affection. All procedures followed the same surgical approach as described in the methods.

### Preoperative radiological data

**Figure 1:**
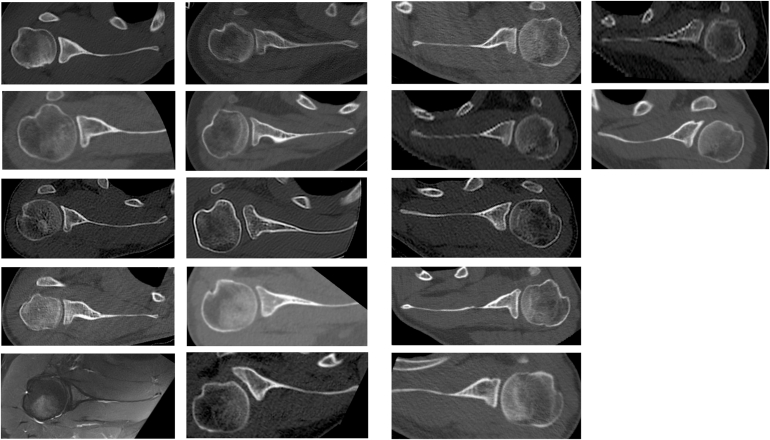
CT/MRI scans of measured affected shoulders. Ten right-side affected presented here, with 9 CT scans and 1 MRI scan, and 7 left-side affected presented with CT scans. *CT*, computed tomography; *MRI*, magnetic resonance imaging.

Preoperative radiological assessments confirmed a spherical humeral head and a concave glenoid in all cases. Imaging was available from 16 computed tomography scans and 1 magnetic resonance imaging scan (Fig. 1). The mean time from imaging to surgery was nine months (range: 4-18 months).

On the affected side, the mean glenoid version was 11.8° of retroversion. Among the 12 patients with bilateral radiological assessments, a significant difference of 4.9° increased retroversion was observed in the affected shoulder compared to the unaffected side (*P* = .022, paired *t*-test; Table I). In the 11 patients with bilateral humeral head version measurements, the affected side demonstrated significantly increased anteversion of 26.3° (*P* = .0006). No significant differences were found in percentage of the humeral head anterior to the midpoint of the glenoid fossa among the 12 patients with bilateral glenoid and humeral head assessments (Table I).

**Table I:** Table of radiological assessments based on preoperative CT/MRI.

Measure (CT/MRI)	n	Affected side, mean (range)	Unaffected side mean (range)	Difference	*P* value
GV all, affected side	17	−11.8° (−32 to −2)	-	-	-
GV bilateral	12	−9.8° (−24 to −2)	−4.9° (−12 to −1)	4.9°	.022
GV only affected side	5	−16.5° (−32 to −10)	-	-	-
PHHA all, affected side	17	42.8%	-	-	-
PHHA bilateral	12	43.9%	46.4%	2.5%	.25
PHHA only affected side	5	40.2%	-	-	-
HHV	11∗	4.5° (−20 to 25)	−21.8° (−49 to 0)	26.3° (4-64)	.0006

*CT*, computed tomography; *MRI*, magnetic resonance imaging; *GV*, glenoid version; *PHHA*, percentage of humeral head anterior to the middle of the glenoid fossa; *HHV*, humeral head version; *n*, number of cases.

For GV and PHHA, the affected side was available in all cases in study = 17. For ‘GV bilateral,’ 12 cases had bilateral available radiological assessment. For ‘GV only affected side,’ 5 cases had radiological assessment only for the affected side. PHHA was assessed as for GV, with the affected side in all 17 cases, bilateral in 12 cases, and thus only one side in 5.

∗ For one patient, not included in the 11, data on humeral head version was only available for the affected side: 30 degrees of anteversion. *P* = paired *t*-test, two-tailed.

### Preoperative and postoperative clinical assessments

Preoperatively, the mean aER of the affected shoulder was −8° (range: −30° to 10°), shoulder abduction was 129° (range: 70°-180°), and the MMS was 18 (range: 16-22). At follow-up at least 1 year postoperatively, aER had significantly improved to a mean of 34° (range: 20°-50°, *P* < .00001), while abduction remained stable at 130° (*P* = .68). The MMS increased to 22 (range: 17-28). The hand-to-neck component of the MMS increased from 2.9 preoperatively to 3.4 postoperatively (*P* = .04). Group mean passive ER were preoperatively −3°, intraoperatively after release and sutured subscapularis tendon 55°, and at final follow up 50°.

Importantly, midline function, as measured by the hand-to-belly component of the MMS, remained stable (preoperative: 3.4; postoperative: 3.5; *P* = .16, see Table II).

**Table II:** Table of preoperative and postoperative clinical assessment on all 17 patients in study.

	Preoperatively	Postoperatively	Difference	*P* value
Active external rotation	−8° (−30 to 10)	34° (20-50)	42°	>.00001
Abduction	129° (70-180)	130° (70-180)	1°	.68
MMS hand-to-belly	3.4 (3-4)	3.5 (3-4)	0.1	.16∗

*MMS*, Modified Mallet Score.

*P* = paired *t*-test, 2 tailed.

∗ Wilcoxon signed-rank test performed due to ordinal data, test statistics .0.

### Functional outcome based on nerve injury level

Patients were categorized by BPBI severity into Erb palsy (C5-6, n = 11), extended Erb palsy (C5-7, n = 4), and global palsy (C5-T1, n = 2). Given the limited sample size in each subgroup, statistical comparisons were not feasible. However, the glenoid retroversion angle in these groups was 11°, 13°, and 14°, respectively. Preoperative vs. postoperative abduction was 150° vs. 151°, 85° vs. 78°, and 110° vs. 115°, respectively. The corresponding gains in aER were 43°, 35°, and 45°, respectively. Despite greater overall functional impairment in patients with extended or global nerve involvement, their gains in aER following surgery appeared comparable to those with less extensive injuries.

### Patient-reported outcome

At a long-term follow-up of at least 2.5 years (mean: 6.0 years, range: 2.5-8 years), patients were asked whether, given their current knowledge of the surgical outcome, they would still choose to undergo the procedure. Fifteen patients (88%) answered ‘*yes*’, while 2 (12%) answered ‘*no*’.

The 2 patients who responded ‘*no*’ reported no perceived positive effects from surgery. One, categorized as having Erb palsy, experienced increased pain with use, while the other, with extended Erb palsy, reported neither positive nor negative effects. Both patients had objectively gained 20° of aER postoperatively.

Among the 15 patients (88%) who responded ‘*yes,*’ all reported an improved ability to move the arm, while 5 indicated that their main benefit was reduced shoulder pain. Another 5 patients reported improved arm posture following surgery. Four patients noted the surgical scar as a negative aspect of the procedure.

### Glenoid version as prognostic factor for the improvement in active external rotation

**Figure 2:**
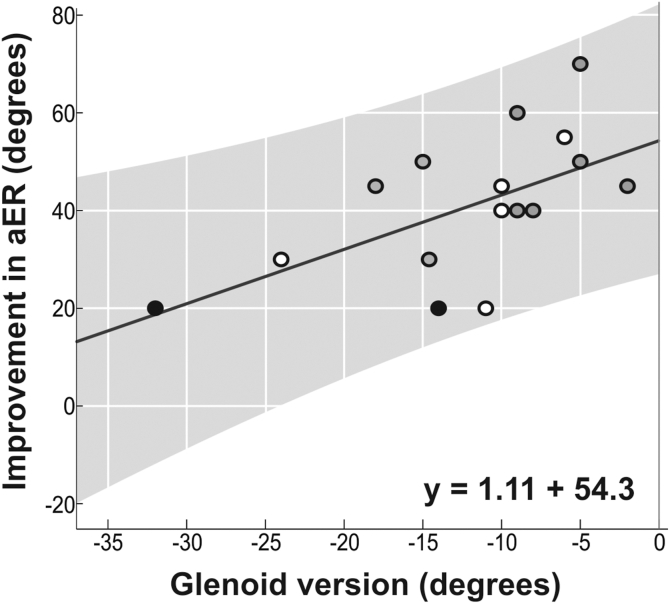
Scatterplot illustrating the relationship between glenoid version and improvement in aER following surgery. The *black regression line* represents the mean expected gain, while the *gray-shaded area* denotes the 95% prediction interval, indicating the expected range of individual improvements in aER for patients with similar preoperative characteristics. The regression model y = 1.11x + 54.3 indicates that for every 1° increase in glenoid version, aER improves by an average of 1.11°. The R^2^ = 0.35 suggests that 35% of the variation in aER gain can be explained by glenoid retroversion. The lower boundary of the prediction interval crosses zero at approximately 24° of glenoid retroversion, indicating a potential threshold beyond which further retroversion may not yield functional gains. In addition, data points are color-coded based on patient-reported outcomes: *black dots*, patients who indicated they would not have opted for surgery knowing the outcome. *Black circles* with gray filling, patients who indicated they would undergo surgery again given the results and reported gain in movement as their primary perceived improvement. *Black circles* with white filling, patients who indicated they would undergo surgery again given the results and reported pain relief as their primary perceived improvement. Note that 2 data points overlap with 40° gain in aER and −8° glenoid retroversion angle. *aER*, active external rotation.

Linear regression analysis revealed a significant association between glenoid retroversion and the improvement in aER following surgery (β = 1.11, *P* = .01, R^2^ = 0.35), indicating that patients with less retroversion exhibited greater improvement in aER (Fig. 2).

## Discussion

This study demonstrates the feasibility and effectiveness of subscapularis tendon step lengthening and partial coracoid resection in adults with BPBI to improve aER without compromising internal rotation (midline function). Our findings also suggest a relationship between glenoid retroversion and aER improvement, with greater retroversion associated with diminished postoperative gains. Notably, the prediction interval introduced in this study provides a valuable tool for estimating surgical outcomes in adult BPBI patients with similar preoperative functional scores.

Patient-reported outcomes revealed that 88% (15/17) of patients were satisfied with their surgical results and would choose surgery again. Interestingly, the 5 patients that cited pain relief as a primary benefit, including 2 who gained only 20° of aER, suggest that improvements in pain and shoulder kinematics may be more meaningful to some patients than absolute aER gains. The reported reduction in pain was primarily described as decreased periscapular discomfort rather than symptoms consistent with subcoracoidal impingement, which none of the patients reported preoperatively. Although improved range of motion after subscapular tendon step lengthening and partial coracoid resection may reduce compensatory scapular muscle loading and thereby lessen periscapular pain, thoracoscapular and GH movements were not evaluated independently in the present study. Further investigation is needed to better understand potential mechanisms of pain reduction following this procedure.

Conversely, 12% (2/17) expressed dissatisfaction, one due to postoperative pain and the other due to no perceived functional improvement, despite both gaining 20° of aER. These patients had greater glenoid retroversion, suggesting that higher retroversion angles may limit surgical benefits. These findings highlight the importance of careful patient selection and preoperative counseling to align expectations with probable outcomes.

While formal statistical analysis was not feasible for patient satisfaction data, qualitative responses underscore that objective functional gains do not always correlate with perceived benefits, emphasizing the need for individualized surgical decision-making.

Variability in aER improvements suggests that multiple factors contribute to surgical success, particularly infraspinatus function and glenoid morphology. The observed negative correlation between glenoid retroversion and aER gain supports the hypothesis that joint architecture influences muscle recruitment and reinnervation potential.

The role of infraspinatus reinnervation remains an open question. While BPBI can lead to permanent infraspinatus denervation, our findings suggest that some patients retain residual neuromuscular function, which may be unmasked and/or enhanced following contracture release. However, patients with greater glenoid retroversion exhibited lower aER gains, implying that altered joint biomechanics may limit infraspinatus activation and compromise surgical outcomes. Future research incorporating electromyographic analysis could provide more definitive insights into infraspinatus function and its contribution to postoperative aER improvements.

A major concern with subscapularis tendon lengthening is the potential loss of internal rotation function. In this study, midline function remained stable, as demonstrated by unchanged MMS hand-to-belly scores. This preservation is likely due to the stepwise surgical approach, which maintains proximal subscapularis integrity and allows controlled lengthening while minimizing excessive release. We speculate that preserving the functional integrity of the caudal subscapularis in our surgical technique may contribute to this outcome, though further studies are needed to confirm this hypothesis.

Despite its valuable insights, this study has several limitations. First, while widely used, the MMS is a relatively crude tool for assessing shoulder function. Advanced motion analysis techniques or 3D kinematic assessments could provide a more detailed understanding of postoperative improvements,^12^^,^^23^ including the relative contributions of GH and scapulothoracic (ST) motion. Such techniques were not available for the present cohort, and we were therefore unable to determine whether the observed gain in aER resulted primarily from increased GH excursion, compensatory ST motion, or a combination of both. Nevertheless, the absence of deterioration in midline/internal rotation function suggests that any contribution from ST motion did not compromise global internal rotation. Future prospective studies using high-resolution kinematic assessment will be important to clarify joint-specific mechanisms of improvement and the biomechanical effects of subscapularis lengthening in adults with BPBI.

The small sample size reduces statistical power for subgroup analyses. Furthermore, while we identified a correlation between glenoid retroversion and postoperative aER gains, causation cannot be inferred. Prospective studies with larger cohorts and control groups are needed to validate these findings.

This study includes patients with preserved GH joint morphology, representing a distinct BPBI subpopulation. However, the etiology of joint deformities in BPBI remains unclear—whether primarily dictated by nerve injury severity, secondary muscle imbalances, or perinatal joint trauma. Understanding these factors is critical for refining patient selection criteria and optimizing surgical decision-making.

A key contribution of this study is the introduction of a prediction interval for surgical prognosis (Fig. 2). Unlike confidence intervals, which estimate mean expected outcomes, the prediction interval accounts for individual variability, offering a clinically relevant tool for patient counseling. For example, the lower boundary of the prediction interval suggests that patients with glenoid retroversion >24° may have a limited likelihood of achieving meaningful aER improvement, reinforcing the need for careful preoperative assessment. Whether this model is applicable to other BPBI subgroups, including pediatric patients, remains an open question for future research.

## Conclusion

Our findings show that subscapularis tendon step lengthening and partial coracoidectomy in previously untreated adults with BPBI and congruent shoulder joints effectively improves aER without compromising midline function. In addition, glenoid retroversion negatively correlates with postoperative aER improvement, suggesting that joint morphology plays a key role in surgical success. These insights have important implications for patient selection, surgical planning, and long-term functional expectations in BPBI management.

More broadly, these results challenge the notion that adult BPBI patients with limited aER cannot benefit from surgical intervention. This underscores the need for greater awareness among health care professionals, including general practitioners and physiotherapists, to identify suitable surgical candidates. Expanding education and outreach initiatives may improve patient identification and referral pathways, ultimately increasing access to surgical intervention and optimizing functional outcomes for adults with BPBI.

## Disclaimers

Funding: Financial support enabling this research was provided through a regional agreement between 10.13039/501100004885Umeå University and 10.13039/501100002960Västerbotten County Council (ALF, Clinical research Grant No.1013248).

Conflicts of interest: The authors, their immediate families, and any research foundations with which they are affiliated have not received any financial payments or other benefits from any commercial entity related to the subject of this article.

## References

[1] Abdelgawad A.A., Pirela-Cruz M.A. (2014). Humeral rotational osteotomy for shoulder deformity in obstetric brachial plexus palsy: which direction should I rotate?. Open Orthop J.

[2] Al-Qattan M.M. (2003). Classification of secondary shoulder deformities in obstetric brachial plexus palsy. J Hand Surg Br.

[3] Bertelli J.A. (2009). Lengthening of subscapularis and transfer of the lower trapezius in the correction of recurrent internal rotation contracture following obstetric brachial plexus palsy. J Bone Joint Surg Br.

[4] Birch R., Birch R.B.G., Wynn Parry C.B. (1998). Surgical disorders of the peripheral nerves.

[5] Birch R. (2000). The growing hand.

[6] Birch R., Gilbert A. (2001). Brachial plexus injuries.

[7] van de Bunt F., Pearl M.L., van Noort A. (2020). Humeral retroversion (complexity of assigning reference axes in 3D and its influence on measurement): a technical note. Strateg Trauma Limb Reconstr.

[8] Burns H.R., Moreno T.A., McLennan A.L., Xue E.Y., Nguyen J.L., Moore B.K. (2024). Surgical technique: spinal accessory to infraspinatus nerve transfer in brachial plexus birth injury. Tech Hand Up Extrem Surg.

[9] Dahlin L.B., Erichs K., Andersson C., Thornqvist C., Backman C., Duppe H. (2007). Incidence of early posterior shoulder dislocation in brachial plexus birth palsy. J Brachial Plex Peripher Nerve Inj.

[10] Friedman R.J., Hawthorne K.B., Genez B.M. (1992). The use of computerized tomography in the measurement of glenoid version. J Bone Joint Surg Am.

[11] Grahn P., Poyhia T., Nietosvaara Y. (2023). Permanent brachial plexus birth injury: helsinki shoulder protocol. Semin Plast Surg.

[12] Grip H., Kallstromer A., Ohberg F. (2022). Validity and reliability of wearable motion sensors for clinical assessment of shoulder function in brachial plexus birth injury. Sensors (Basel).

[13] Hoeksma A.F., ter Steeg A.M., Nelissen R.G., van Ouwerkerk W.J., Lankhorst G.J., de Jong B.A. (2004). Neurological recovery in obstetric brachial plexus injuries: an historical cohort study. Dev Med Child Neurol.

[14] Hoeksma A.F., Wolf H., Oei S.L. (2000). Obstetrical brachial plexus injuries: incidence, natural course and shoulder contracture. Clin Rehabil.

[15] Hui J.H., Torode I.P. (2003). Changing glenoid version after open reduction of shoulders in children with obstetric brachial plexus palsy. J Pediatr Orthop.

[16] Johansson A., Paul U., Anna-Lena L. (2019). Obstetric brachial plexus palsy - a prospective, population-based study of incidence, recovery and long-term residual impairment at 10 to 12 years of age. Eur J Paediatr Neurol.

[17] Jonsson K., Werner M., Roos F., Hultgren T. (2019). Development of the glenohumeral joint after subscapular release and open relocation in children with brachial plexus birth palsy: long-term results in 61 patients. J Shoulder Elbow Surg.

[18] Kozin S.H. (2011). The evaluation and treatment of children with brachial plexus birth palsy. J Hand Surg Am.

[19] Manske M.C., Kalish L.A., Cornwall R., Peljovich A.E., Bauer A.S. (2020). Reconstruction of the suprascapular nerve in brachial plexus birth injury: a comparison of nerve grafting and nerve transfers. J Bone Joint Surg Am.

[20] Nath R.K., Melcher S.E., Paizi M. (2006). Surgical correction of unsuccessful derotational humeral osteotomy in obstetric brachial plexus palsy: evidence of the significance of scapular deformity in the pathophysiology of the medial rotation contracture. J Brachial Plex Peripher Nerve Inj.

[21] Nikolaou S., Peterson E., Kim A., Wylie C., Cornwall R. (2011). Impaired growth of denervated muscle contributes to contracture formation following neonatal brachial plexus injury. J Bone Joint Surg Am.

[22] Pearl M.L., Edgerton B.W., Kon D.S., Darakjian A.B., Kosco A.E., Kazimiroff P.B. (2003). Comparison of arthroscopic findings with magnetic resonance imaging and arthrography in children with glenohumeral deformities secondary to brachial plexus birth palsy. J Bone Joint Surg Am.

[23] Russo S.A., Nice E.M., Chafetz R.S., Richards J.G., Zlotolow D.A., Kozin S.H. (2025). Impact of tendon transfer on scapulothoracic and glenohumeral motion in children with brachial plexus birth injuries. J Shoulder Elbow Surg.

[24] Soldado F. (2020). Double nerve transfer for restoring external rotation of the glenohumeral joint after neonatal brachial plexus injury. Microsurgery.

[25] Soldado F., Rojas-Neira J., Gonzalez-Morgado D., De Avila-Diaz I., Levaro-Pano F., Bertelli J.A. (2025). Very late spinal accessory nerve to infraspinatus nerve transfer to restore glenohumeral external rotation in brachial plexus birth injury. Plast Reconstr Surg.

[26] Sommarhem A.J., Grahn P.M., Nietosvaara Y.A. (2015). Selective neurotization of the infraspinatus muscle in brachial plexus birth injury patients using the accessory nerve. Plast Reconstr Surg.

[27] Van Heest A., Glisson C., Ma H. (2010). Glenohumeral dysplasia changes after tendon transfer surgery in children with birth brachial plexus injuries. J Pediatr Orthop.

[28] Waters P.M., Bae D.S. (2008). The early effects of tendon transfers and open capsulorrhaphy on glenohumeral deformity in brachial plexus birth palsy. J Bone Joint Surg Am.

[29] Waters P.M., Smith G.R., Jaramillo D. (1998). Glenohumeral deformity secondary to brachial plexus birth palsy. J Bone Joint Surg Am.

